# Organizing empyema induced in mice by *Streptococcus pneumoniae*: effects of plasminogen activator inhibitor-1 deficiency

**DOI:** 10.1186/s40169-016-0097-2

**Published:** 2016-05-13

**Authors:** Torry A. Tucker, Ann Jeffers, Jake Boren, Brandon Quaid, Shuzi Owens, Kathleen B. Koenig, Yoshikazu Tsukasaki, Galina Florova, Andrey A. Komissarov, Mitsuo Ikebe, Steven Idell

**Affiliations:** The Department of Cellular and Molecular Biology, The University of Texas Health Science Center at Tyler, 11937 US HWY 271, Biomedical Research Building, Lab C-5, Tyler, TX 75708 USA

**Keywords:** Pleural mesothelial cells, Pneumonia, Plasminogen activator inhibitor-1

## Abstract

**Background:**

Pleural infection affects about 65,000 patients annually in the US and UK. In this and other forms of pleural injury, mesothelial cells (PMCs) undergo a process called mesothelial (Meso) mesenchymal transition (MT), by which PMCs acquire a profibrogenic phenotype with increased expression of α-smooth muscle actin (α-SMA) and matrix proteins. MesoMT thereby contributes to pleural organization with fibrosis and lung restriction. Current murine empyema models are characterized by early mortality, limiting analysis of the pathogenesis of pleural organization and mechanisms that promote MesoMT after infection.

**Methods:**

A new murine empyema model was generated in C57BL/6 J mice by intrapleural delivery of *Streptococcus pneumoniae* (D39, 3 × 10^7^–5 × 10^9^ cfu) to enable use of genetically manipulated animals. CT-scanning and pulmonary function tests were used to characterize the physiologic consequences of organizing empyema. Histology, immunohistochemistry, and immunofluorescence were used to assess pleural injury. ELISA, cytokine array and western analyses were used to assess pleural fluid mediators and markers of MesoMT in primary PMCs.

**Results:**

Induction of empyema was done through intranasal or intrapleural delivery of *S. pneumoniae*. Intranasal delivery impaired lung compliance (p < 0.05) and reduced lung volume (p < 0.05) by 7 days, but failed to reliably induce empyema and was characterized by unacceptable mortality. Intrapleural delivery of *S. pneumoniae* induced empyema by 24 h with lung restriction and development of pleural fibrosis which persisted for up to 14 days. Markers of MesoMT were increased in the visceral pleura of *S. pneumoniae* infected mice. KC, IL-17A, MIP-1β, MCP-1, PGE_2_ and plasmin activity were increased in pleural lavage of infected mice at 7 days. PAI-1^−/−^ mice died within 4 days, had increased pleural inflammation and higher PGE_2_ levels than WT mice. PGE_2_ was induced in primary PMCs by uPA and plasmin and induced markers of MesoMT.

**Conclusion:**

To our knowledge, this is the first murine model of subacute, organizing empyema. The model can be used to identify factors that, like PAI-1 deficiency, alter outcomes and dissect their contribution to pleural organization, rind formation and lung restriction.

## Background

Pleural infection remains a common and important clinical problem that can complicate pneumonia or occur after trauma. The incidence of empyema continues to rise despite the broad use of vaccinations and development of more potent antibiotics [1, 2]. Of the roughly 4 million cases of pneumonia annually diagnosed in the US, about half develop a parapneumonic effusion [1]. In many cases, these effusions can evolve with formation of complicated parapneumonic effusions or frank empyema, which is characterized by overt infection or intrapleural pus. Biomarkers such as low pH predict the development of pleural loculation [1]. About 65,000 patients in the US and UK suffer from pleural infection each year [1, 3], which is associated with increased morbidity, mortality and medical costs approaching half a billion dollars annually [4, 5]. About 40,000 US patients annually may require pleural drainage to prevent morbidity associated with complicated parapneumonic effusions/empyema in the US each year [6]. Current surgical treatment is invasive and alternative treatment with intrapleural administration of fibrinolysins is associated with variable outcomes in adults [7]. These considerations justify the search for more effective interventions, which rely on better understanding of the pathogenesis of pleural organization and remodeling [8].

In a previous publication, we showed that a combination of bleomycin and carbon black (CBB) could reliably and reproducibly induce pleural injury including reduced lung function and increased pleural thickening in C57BL/6 mice [9]. We also reported a *Pasteurella multocida* model of pleural injury in rabbits [10]. Although this injury is quite robust, simulates human pleural injury and has been used to test the efficacy of fibrinolytic agents, this pathogen rarely causes pleural infection in clinical practice. Because the use of larger animals limits use of transgenic or knock-out animals, a murine model of pleural injury and repair in the C57BL/6 strain is desirable. While a previous study showed that intranasally administered *S. pneumoniae* (D39 strain) produced intrapleural injury in CD1 mice, that model was characterized by early mortality that restricted analyses to the acute setting; over 48 h [11]. Pleural infection was lethal thereafter, precluding analysis of the remodeling that occurred after acute injury. To our knowledge, no murine model of empyema and progressive pleural organization after bacterial infection has been reported, which has slowed progress in the field. We inferred that a more durable model of infectious pleural injury could be developed and tested that postulate using C57BL/6 mice to develop a model of organizing of *S. pneumoniae* pleural empyema with survivorship over 2 weeks. We used the model to assess the impact of PAI-1 deficiency on the evolution of organizing empyema, as this derangement was previously reported to increase pleural rind formation and lung restriction in the CBB model of noninfectious pleural injury [9].

## Methods

### Intranasal and intrapleural inoculations

All experiments involving animals were approved by the Institutional Animal Care and Use Committee at the University of Texas Health Science Center at Tyler. C57BL/6 J mice (10–12 weeks of age, ≈20 g, Jackson Laboratory, Bar Harbor ME) were lightly anesthetized with isoflurane. Intranasal inoculations (3 × 10^7^–5 × 10^9^) of *Streptococcus pneumoniae* (*S. pneumoniae,* D39, National Collection of Type Cultures, Salisbury UK) resuspended in 0.9 % saline were delivered in 40 µl over the nares. Intrapleural inoculations (5 × 10^7^–5 × 10^8^ cfu, resuspended in 0.9 % saline) of *S. pneumoniae* were delivered in 150 µL by intrapleural injection. The control group received normal saline under the same conditions. Antibiotic treatment (enrofloxacin, 15 mg/kg) was initiated 18 h post infection and was administered daily by subcutaneous injection for 4 days. Mice were periodically monitored following infection to record body weight, dehydration status, activity and behavior. If dehydration was detected by alterations of skin turgor, the affected mice were subcutaneously injected with 200–500 µl of warmed 0.9 % saline, as needed. Moribund animals were euthanized. After administration of *S. pneumoniae*, mice were housed on a heating pad to maintain an ambient temperature of 30 °C throughout the time course.

### Lung and pleural lavage collection

Lung and pleural lavages were performed using 1.5 ml of sterile normal saline were immediately performed at the time of death in selected animals, as previously described [9]. Total white cell and differential cell counts were likewise measure in these fluids, as we previously reported [9].

### Cultures of pleural fluids

Pleural lavages of saline and *S. pneumoniae* infected mice were cultured on blood agar plates containing 5 % sheep blood (Remel Blood Agar, Fisher Scientific). Neat (50 µl) and 1:100 dilutions of the pleural lavages were cultured on blood agar plates and incubated 15 h at 37 °C. Colonies were then counted to determine bacterial burden.

### Lung histology, immunostaining, confocal, bright field microscopy and morphometry

Lung histology and immunostaining were performed as previously described [9, 12]. All tissue sections were first deparafinized and subjected to antigen retrieval using a citrate buffer at 95 °C for 20 min. Tissue analyses, collagen deposition and localization were initially assessed by Trichrome staining as previously described [9, 12]. Morphometric analyses of pleural tissue thickness and depth of underlying pneumonitis were performed as we previously described [9]. Fibrin (ogen) antigen was assessed using immunohistochemistry (IHC) and Fast Red (BioGenex, Freemont CA) chromogen as previously described [13].

Immunofluorescence was used to visualize α-SMA and calretinin expression in saline and *S. pneumoniae* infected pleuropulmonary sections as previously described [9]. Confocal microscopy was then used to visualize immunofluorescence and co-localization of the markers. Confocal images were acquired from a field of view at 0.4-µm z-axis increments with the LSM 510 Meta confocal system (Carl Zeiss) at 40× as previously described [9, 13].

### Collagen detection in lung tissues

Bright field microscopy was used to image trichrome stained tissue sections as previously described [9]. Collagen was detected by picrosirius staining and imaged using a polarized light source on a Nikon Ti inverted microscope.

### Pulmonary function testing

Pulmonary function tests were performed immediately before CT imaging and prior to sacrifice, as previously described [9, 12]. Briefly, mice were anesthetized with a ketamine/xylazine mixture. Anesthetized mice were intubated by inserting a sterile, 20-gauge intravenous cannula through the vocal cords into the trachea. Measurements were then performed using the flexiVent system (SCIREQ, Tempe AZ). The “snapshot perturbation method” was used to determine lung compliance, according to manufacturer’s specifications. Mice were maintained under anesthesia using isoflurane during pulmonary function testing.

### Computed tomography (CT) scans and measurements of lung volume

Chest CT imaging and measurements of lung volume were performed as previously described [9, 12]. Ketamine/xylazine anesthetized mice were anesthetized further using an isoflurane/oxygen mixture to minimize spontaneous breaths and to ensure that mice remained anesthetized throughout the procedure. Images were obtained using the Explore Locus Micro-CT Scanner. CT scans were performed during full inspiration and at a resolution of 93 µm. Microview software was used to analyze lung volumes and render three-dimensional images. Lung volumes were calculated from renditions collected at full inspiration.

### Measurement of pleural fluid plasmin and fibrinolytic activity

Plasmin activity in the pleural washes of WT and PAI-1^−/−^ mice treated with saline or *S. pneumoniae* was measured by amidolytic assay using a plasmin substrate (PL-5268, Centerchem Inc, Norwalk CT) on a SpectraMax 96-well optical absorbance plate reader (Molecular devices, Sunnyvale, CA), as previously described [14]. Fibrinolytic activity was measured as previously described [14].

### Bioplex analyses of pleural lavage mediators

Analyses of pleural lavage inflammatory mediators including: Eotaxin, G-CSF,GM-CSF,IFN-γ, IL-1α, IL-1β, IL-2, IL-3, IL-4, IL-5, IL-6, IL-9, IL-10, IL-12 (p40), IL-12 (p70), IL-13, IL-17A, KC, MCP-1 (MCAF), MIP-1α, MIP-1β, RANTES, TNF-α were determined using the Bio-Plex Pro Mouse Cytokines 23-plex (BIO-RAD) on a BioPlex MAGPIX Multiplex Reader according to the manufacturer’s instructions.

### PGE_2_ ELISA

PGE_2_ was first extracted from pleural lavage by C18 column purification according to manufacturer’s directions (Cayman Chemical, Ann Arbor Michigan). PGE_2_ levels were determined by competitive ELISA (Cayman) according to manufacturer’s instructions.

### Primary pleural mesothelial cell culture and treatment

Permission to collect and use HPMCs was granted through an exempt protocol approved by the Institutional Human Subjects Review Board of the University of Texas Health Science Center at Tyler. HPMCs were isolated from pleural fluids collected from patients with congestive heart failure or post-coronary bypass pleural effusions as previously described [15]. HPMCs were maintained in LHC-8 culture media (Life Technologies, Carlsbad CA) containing 3 % fetal bovine serum (Life Technologies), 2 % antibiotic–antimycotic (Life Technologies) and Glutamax (Life Technologies) as previously described [9, 13, 15, 16]. MPMCs were isolated and cultured as previously reported [9]. All cells were cultured in a humidified incubator at 37 °C in 5 % CO_2_/95 % air. Cells were passaged a maximum of five times before discontinuing use. Serum-starved cells were treated with TGF-β (5 ng/ml), PGE_2_ (1 µM), butaprost (EP2 agonist, 1 µM), sulprostone (EP3 agonist, 1 µM) and Cay10598 (EP4 agonist, 1 µM, Cayman). Cell lysates were then Western blotted for α-SMA and β-actin as previously described [9, 17].

### Statistics

All statistics were performed using the Mann–Whitney U test. A *p* value of less than 0.05 was considered significant.

## Results

### Intranasal administration of *S. pneumoniae* did not reliably induce survivable empyema

**Fig. 1:**
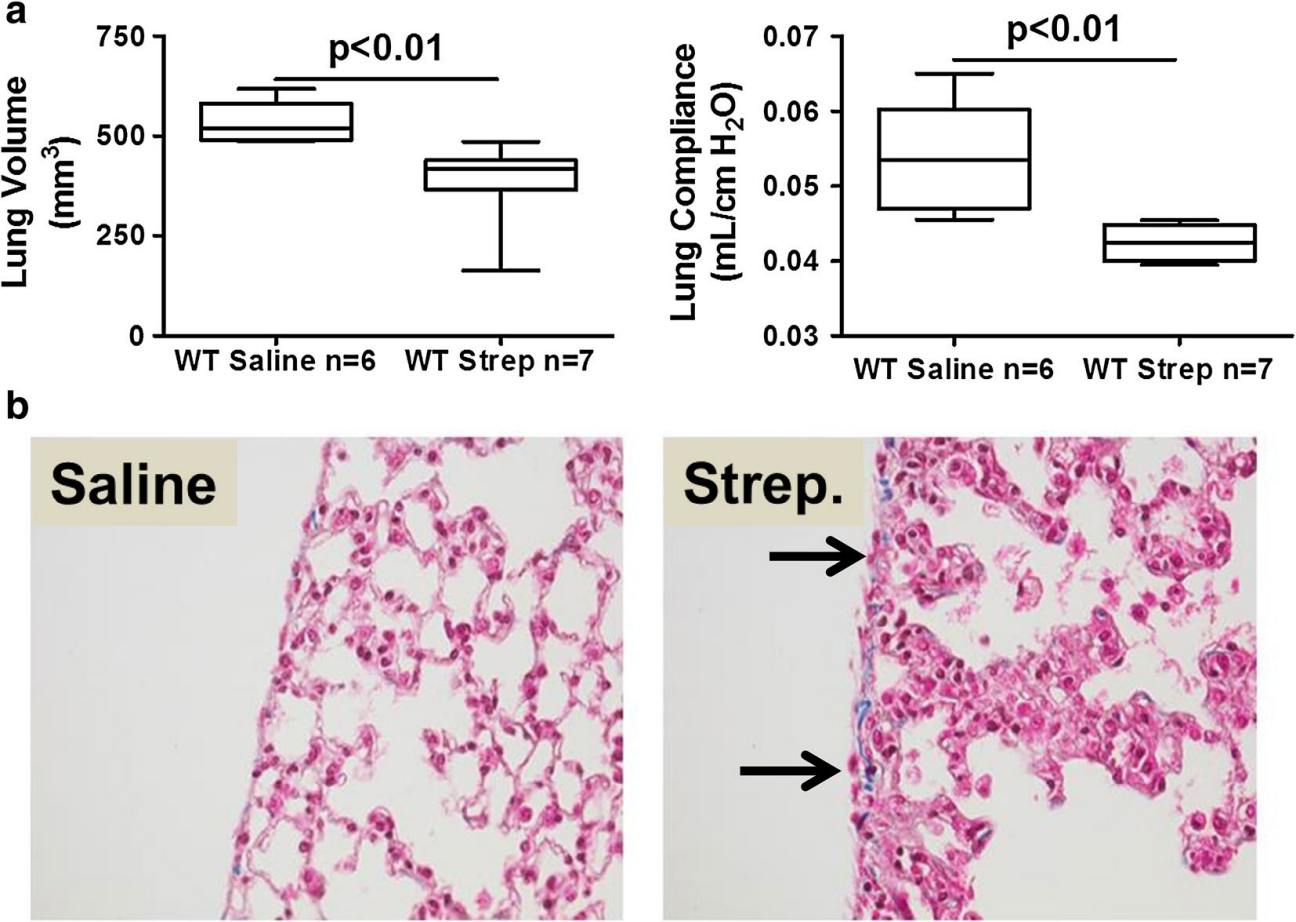
Intranasal *S. pneumoniae* causes pneumonia, lung dysfunction and mild pleural inflammation at survivable intranasal dosing. *S. pneumoniae* (3 × 10^8^CFU) was intranasally administered to WT mice and maintained for 7 days. **a** Lung volumes were measured by CT scan and pulmonary function (compliance) measured as described in the Methods. n = 6–7 mice/group. **b** Peripheral lung tissue sections from saline-challenged and *S. pneumoniae* infected mice collected at 7 days were stained with trichrome to show changes in lung architecture and collagen deposition (*blue stain*). *S. pneumoniae* infected mice exhibited thickened alveolar septae, mild pleural inflammation, reactive visceral mesothelial cells and mildly increased collagen deposition compared to saline controls. No pleural fluid or fibrinous strands at the pleural surface were seen. *Solid arrows* indicate reactive mesothelial cells. Images are 40× and are representative of the findings of 30 fields/slide and 6–7 mice/group

To create a model of pneumonia complicated by empyema, we initially challenged C57BL/6 mice with intranasal administration of *S. pneumoniae*. A range of doses were used to induce empyema in dose–response analyses (3 × 10^7^–5 × 10^9^ cfu). Intranasal administration of *S. pneumoniae* (3 × 10^8^ CFU) significantly reduced lung volume (Fig. 1a, p < 0.01) and decreased compliance (p < 0.01). As previously reported [11], intranasal administration of *S. pneumoniae* was associated with significant mortality without antibiotics (data not shown), so that a quinolone to which the organism is exquisitely susceptible was begun at 18 h. Doses of less than 3 × 10^8^ cfu did not induce detectable changes in lung function. A range of higher intranasal doses (up to 10^9^ CFU) consistently caused early mortality (<24 h) associated with severe diffuse pneumonitis, pleural inflammation and pleural effusions and were not further investigated (data not shown). Tissue sections from saline and infected mice were next analyzed to assess changes in lung histology and collagen deposition. Alveolar inflammation was apparent at 7 days after 3 × 10^8^ CFU and mild reactive changes of the visceral pleurae were consistently observed (Fig. 1b). Pleural thickness was not significantly increased by intranasal *S. pneumoniae* infection (data not shown). No pleural effusions or adhesions were found at 7 days in any of these animals, indicating that pleural injury was relatively mild in animals treated with a maximal, survivable intranasal dose of the organism.

### Intrapleural administration of *S. pneumoniae* induces pleural organization and remodeling with rind formation

**Fig. 2:**
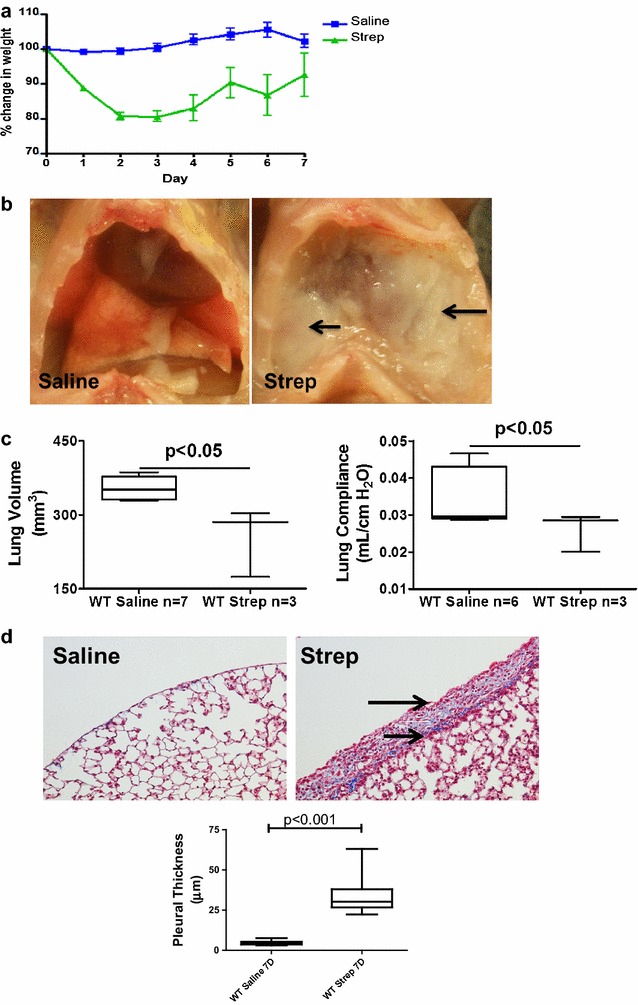
Intrapleural *S. pneumoniae* induces pleural injury and lung dysfunction. *S. pneumoniae* was intrapleurally administered and incubated for 7 days. **a** Weights were collected over 7 days (n = 3–7 mice/group). **b** Gross images taken at 7 days after administration of saline or *S. pneumoniae*. These animals did not receive antibiotic treatment. *S. pneumoniae* infection promotes extensive pleural injury with deposition of a transitional intrapleural fibrinous neomatrix that is readily appreciated with coating and encasement of the lungs. *Solid arrows* indicate areas of neomatrix deposition within the thoracic cavity of *S. pneumoniae* infected mice. **c** Lung volumes and function (compliance) were significantly reduced by *S. pneumoniae* administration at 7 days (p < 0.05). **d** Lung tissue sections from saline-treated and *S. pneumoniae* infected mice (7 days post-infection) were trichrome stained, with collagen deposition indicated by the *blue stain*. By morphometry, *S. pneumoniae* infected mice demonstrated significantly more pleural thickening than saline controls (p < 0.001). *Solid arrows* indicate the pleural surface and the basement membrane of the thickened pleura. Areas of collagen deposition were observed within the thickened pleural rind of *S. pneumoniae* infected mice. Images were obtained at 20× and are representative of the finding of 30 fields/slide and 3–7 mice/group

Because of the lack of pleural organization with sublethal doses of intranasally administered *S. pneumoniae*, we next sought to determine if intrapleural administration yielded survivable empyema. *S. pneumoniae* was therefore injected directly into the pleural space, at a range of doses (5 × 10^7^–5 × 10^8^ cfu) with no antibiotic coverage. We found that doses of less than 1 × 10^8^ CFU did not reliably induce pleural injury, while doses above 3 × 10^8^ cfu were associated with unacceptable early mortality of almost all animals (data not shown). *S. pneumoniae* infected mice demonstrated dramatic decrements in weight for the first 3 days after infection (Fig. 2a). By 7 days, the infected mice had regained most of the lost weight. In initial analyses, mice were euthanized 7 days after *S. pneumoniae* administration. Gross analyses of infected mice showed increased deposition of transitional fibrinous adhesions in the pleural space (Fig. 2b) and the pleural surface was coated with what grossly appeared to be purulent material by 2 days (data not shown). This injury progressively worsened over 7 days, with pleural thickening detectable by 5 days (not shown) and increased by 7 days, often with areas of discrete pleurodesis. As anticipated based on the gross findings, intrapleural administration of *S. pneumoniae* significantly impaired lung volume (p < 0.05) and lung function; compliance (p < 0.05) by 7 days (Fig. 2c). By histologic analysis, infected mice exhibited extensive remodeling characterized by significant pleural thickening with collagen deposition (blue stain) at 7 days compared to saline-treated controls (Fig. 2d). Ultimately, we found that this model was limited by mortality of greater than half of the animals by 7 days, which was unacceptable.

### Antibiotic treatment improves survival of mice intrapleurally infected with *S. pneumoniae*

**Fig. 3:**
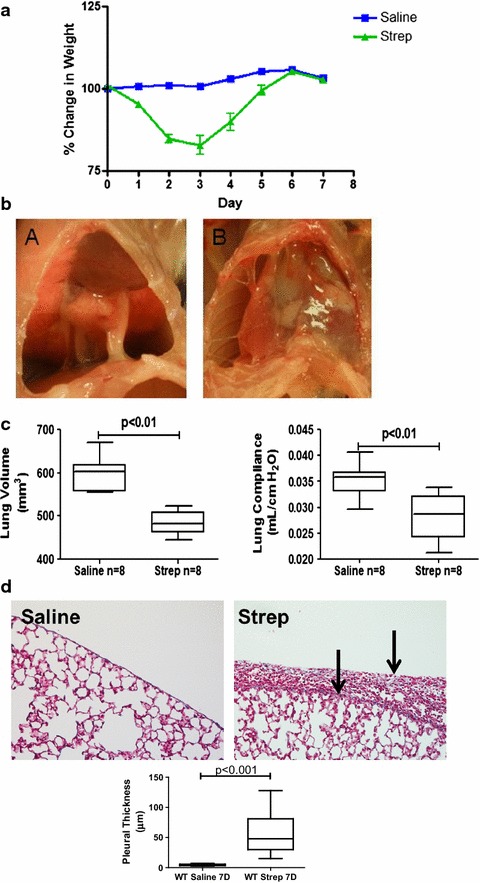
Antibiotic treatment extends survival after *S. pneumoniae*-induced empyema which is characterized by persistent pleural fibrosis, rind formation and lung restriction. Antibiotic treatment was begun 18 h after *S. pneumoniae* was intrapleurally administered. **a** Weights were collected over a 7 days time course (n = 8 animals/group). The infected mice recovered lost weight by 6 days. **b** Gross images were taken 7 days after intrapleural administration of saline or *S. pneumoniae*. *S. pneumoniae* infection promotes progressive pleural injury and deposition of a fibrinous matrix with antibiotic treatment (*subpanel B*) when compared to saline controls (*subpanel A*). Representative images are shown (n = 6–8 animals/group). **c** Lung volumes and function (compliance) were significantly reduced by *S. pneumoniae* infection by 7 days (P < 0.01). **d** Lung tissue sections from saline and *S. pneumoniae* infected mice were trichrome stained for collagen deposition (*blue*). *Solid arrows* indicate the pleural surface and the basement membrane of the thickened pleura. Areas of collagen deposition were observed within the thickened pleural rind of *S. pneumoniae* infected mice. *S. pneumoniae*-infected mice had significantly increased pleural thickening when compared to saline controls (6–8 mice/group)

To expand the experimental time frame so that pleural organization and remodeling could be interrogated over 7–14 days, intrapleurally infected mice were given an antibiotic; enrofloxacin, 18 h after intrapleural administration of *S. pneumoniae* (1.8 × 10^8^). Antibiotic-treated *S. pneumoniae*-infected mice demonstrated rapid weight loss by 3 days after infection (Fig. 3a), similar to mice without antibiotic treatment. However, these mice began to gain weight by 4 days and approached saline control levels by 6 days. Gross analyses (Fig. 3b) showed pronounced pleural injury in antibiotic-treated, *S. pneumoniae* infected mice at 7 days (panel b) when compared to saline treated mice (panel a).

**Fig. 4:**
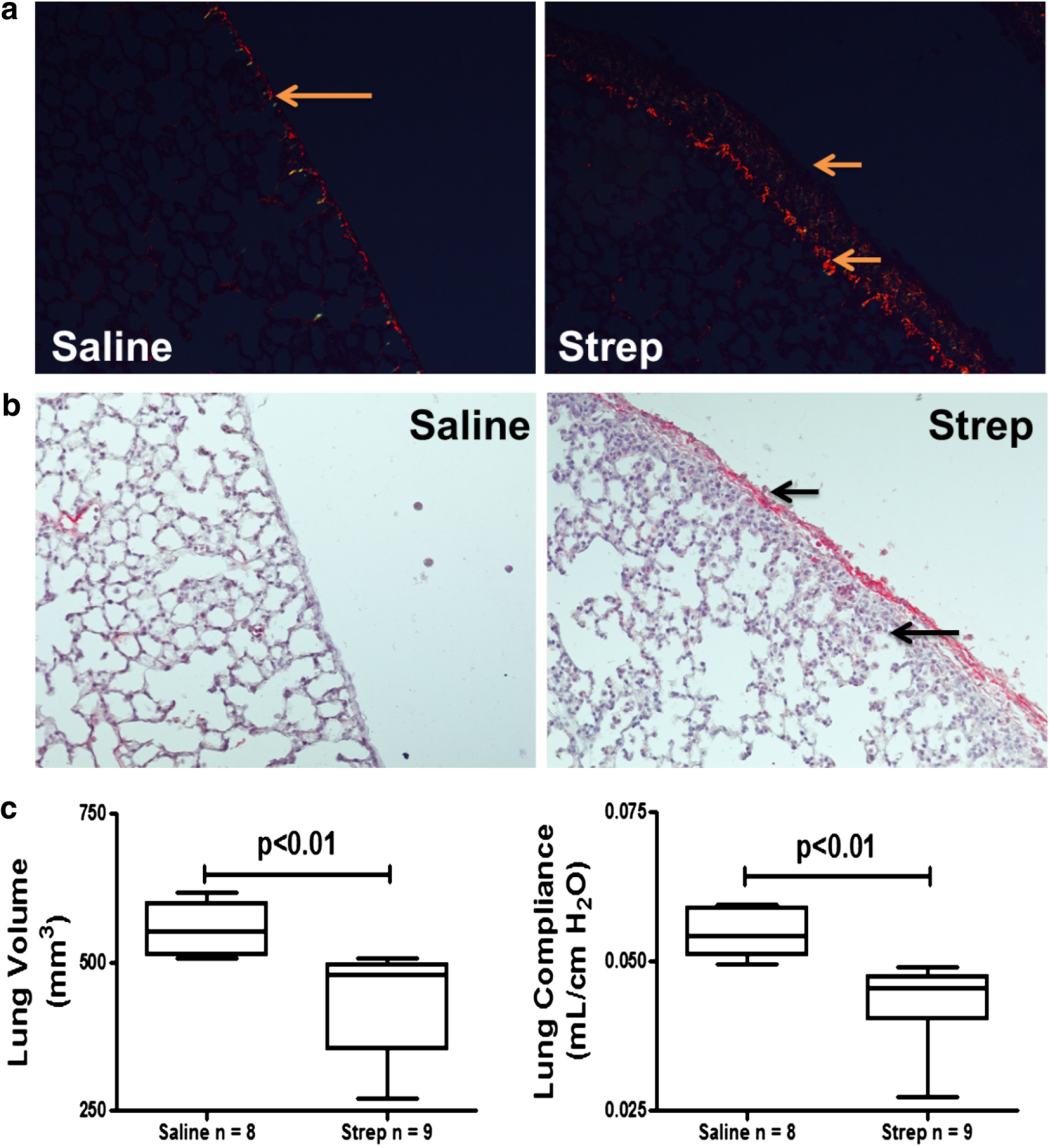
*S. pneumoniae*-induced empyema is characterized by persistent pleural fibrosis, rind formation and lung restriction at 7 days. Antibiotic treatment was begun 18 h after *S. pneumoniae* was intrapleurally administered. **a** Peripheral lung sections from saline and *S. pneumoniae* infected mice were picrosirius stained to detect collagen deposition (*red–orange* birefringence). *S. pneumoniae* infected mice demonstrated increased collagen deposition at the pleural surface and in the subpleural region compared to saline-treated mice. *Solid arrows* indicate the basement membrane and the visceral pleural surface. Areas of collagen deposition were detected at the basement membrane and within the thickened pleural mesothelium. **b** Tissue sections from 7 days saline and *S. pneumoniae* infected mice were stained for fibrin (ogen) by immunohistochemical analysis (*red stain*). Extravascular fibrin (ogen) deposition is readily apparent after *S. pneumoniae* infection. *Solid arrows* indicate the pleural surface and the basement membrane of the thickened pleura of *S. pneumoniae* infected mice. Fibrin(ogen) deposition was prominent at the pleural surface of *S. pneumoniae* infected mice.** c** Lung volumes and function (compliance) were significantly decreased by *S. pneumoniae* infection by 14 days (p < 0.01)

**Fig. 5:**
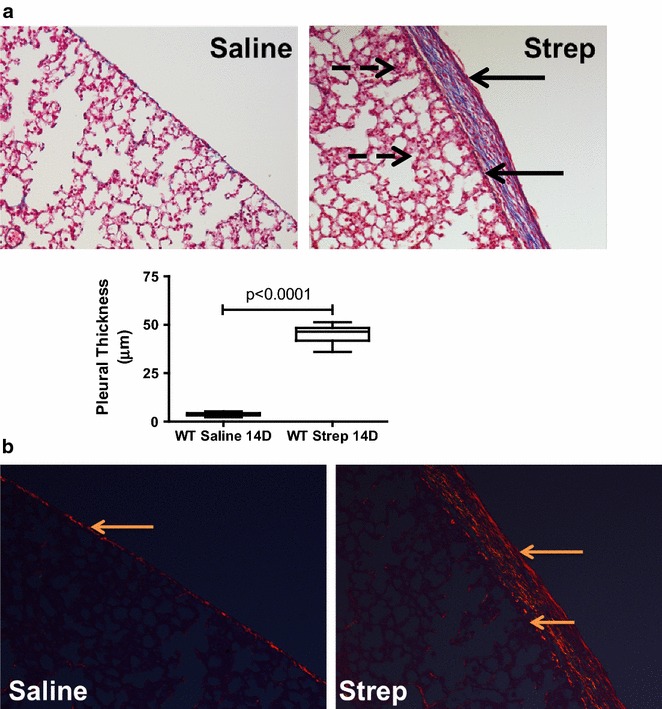
*S. pneumoniae*-induced empyema is characterized by persistent pleural fibrosis, rind formation and lung restriction at 14 days. Antibiotic treatment was begun 18 h after *S. pneumoniae* was intrapleurally administered. **a** Lung tissue sections from saline and *S. pneumoniae*-treated mice were Trichrome stained to assess collagen deposition (*blue*) and determine pleural thickness. 14 days *S. pneumoniae*-infected mice exhibited significantly increased pleural thickening compared to saline controls. *Solid arrows* indicate the pleural surface and the basement membrane of the thickened visceral pleura. *Broken arrows* indicate areas of pneumonitis underlying the thickened pleura. Collagen deposition was detected within the pleural mesothelium of *S. pneumoniae* infected mice. Images were collected at 20× and are representative of 30 fields/slide and 6–8 mice/group. **b** Lung tissue sections from saline and *S. pneumoniae* treated mice were stained with picrosirius to confirm tissue collagen deposition (red–orange birefringence). *S. pneumoniae* infected mice demonstrated increased collagen deposition at the pleural and subpleural space when compared to saline-treated mice at 14 days. *Solid arrows* indicate areas of collagen deposition within the mesothelium of the visceral pleura of saline and *S. pneumoniae* infected mice. Images were taken at 20× and are representative of 30 fields/slide and 6–8 mice/group

We next performed pulmonary function tests and CT scans at 7 days in these mice and found consistent decrements in lung volume (p < 0.01) and pulmonary function; compliance (p < 0.01), versus saline controls (Fig. 3c). Total WBC were significantly elevated (17.7 ± 7.8 × 10^5^ versus 8.6 ± 2.3 × 10^5^ p < 0.01, n = 8) in the pleural lavages of infected mice compared to saline controls at 7 days with antibiotic treatment. The median neutrophil percentage was 25 % at 7 days in the empyema animals and was 1 % in the saline controls. Tissue sections from saline and *S. pneumoniae* infected mice were next analyzed by histology. Because parietal pleural injury was more heterogeneous and responses of the visceral and parietal pleural surfaces were found to be comparable, we focused on determination of changes in the visceral pleura, as we previously reported [9]. *S. pneumoniae* infected mice exhibited pronounced matrix deposition and significant pleural thickening (p < 0.001, Fig. 3d) compared to saline controls. The infection also induced collagen expression in the mesothelium and subpleural mesothelium by 7 days as determined by Picrosirius staining for collagen (red–orange birefringence, Fig. 4a). Tissue sections from saline and *S. pneumoniae*-infected mice were next stained for fibrin(ogen) by immunohistochemistry (red stain, Fig. 4b). Robust fibrin (ogen) deposition was detected at the visceral pleural surface 7 days post-infection. That both fibrin(ogen) and collagen are detectable in the thickened visceral pleura by 7 days after *S. pneumoniae* injury indicates that ongoing pleural organization contributed to the restrictive, physiologic alterations we observed at this time. Pleural lavage from euthanized saline and *S. pneumoniae* infected mice were cultured on blood agar dishes to determine if live *S. pneumoniae* persisted throughout the 7 days time course. With antibiotic treatment, pleural lavages were sterile by 7 days after intrapleural inoculation, while live *S. pneumoniae* were routinely cultured from the lavages of 7 days infected mice that were not treated with antibiotics. In time course experiments, *S. pneumoniae* colonies could not be detected in pleural lavage by 3 days after antibiotic administration (data not shown).

Because antibiotic treatment increased survival of infected mice, we next extended the model over 14 days and determined the effect of *S. pneumoniae* infection on pulmonary function (Fig. 4c). Significant decrements in lung volume (p < 0.01) and pulmonary function; compliance (p < 0.01) persisted at 14 days. Increased collagen deposition (blue stain) and significant increases in pleural thickness remained at 14 days (Fig. 5a). Underlying pneumonitis was detected in *S. pneumoniae* infected mice at 14 days mice. Pleural fibrin deposition and intrapleural adhesions were likewise detectable at this time (data not shown). Collagen deposition, by picrosirius staining, persisted in 14 days infected WT mice compared to saline controls (Fig. 5b). Pleural thickening did not significantly change between 7 and 14 days.

### Mediator Profile in pleural fluids of *S. pneumoniae* infected mice

**Fig. 6:**
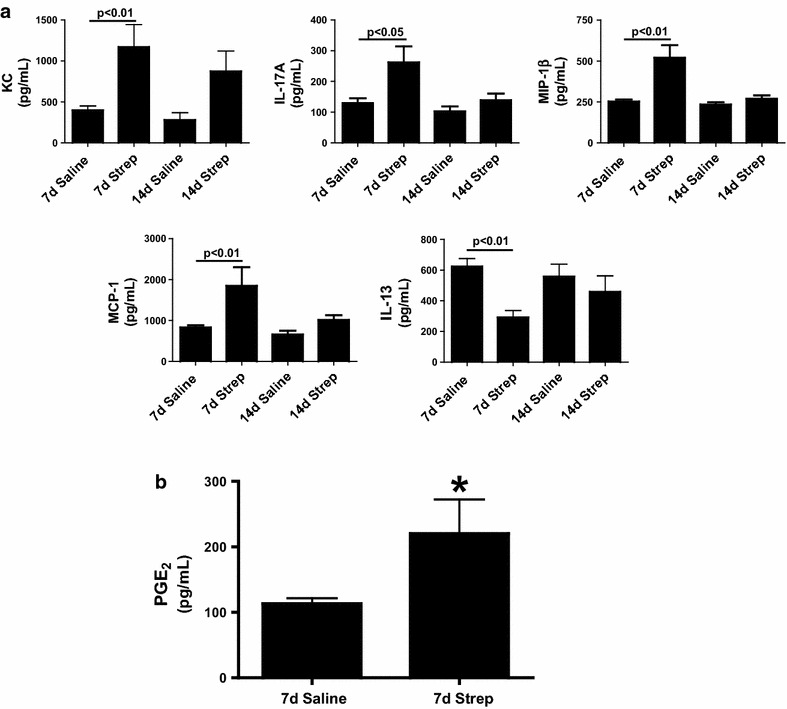
Inflammatory cytokine profile of *S. pneumoniae* infected mice. Lavages were isolated from the pleural cavities of saline and intrapleurally infected *S. pneumoniae* infected mice 7 and 14 days post infection. **a** Proinflammatory mediator expression was then assayed in the collected lavages by Bioplex assay. Pleural lavages showed significant changes in KC, IL-17A, MCP-1, MIP-1β, and IL-13 at 7 days by Bioplex analyses (P < 0.01). n = 6–8 mice/group. PGE_2_ was extracted from the PLs of saline and *S. pneumoniae*-infected mice. PGE_2_ levels were then determined by competitive ELISA. **b** PGE_2_ expression was significantly increased in the lavages of *S. pneumoniae* infected WT mice at 7 days (*denotes a P < 0.05), n = 6–8 mice/group

We next sought to characterize the profile of locally elaborated mediators promoting significant increases in WBC that were observed in pleural fluids of infected mice. We first measured changes in inflammatory mediators. KC, IL-17A, MIP-1β and MCP-1 were significantly increased with *S. pneumoniae* infection by 7 days (p < 0.05) but were not significantly different from control levels by 14 days (Fig. 6a). Conversely, IL-13, an anti-inflammatory cytokine was significantly down-regulated (p < 0.01) in *S. pneumoniae*-infected mice by 7 days but returned to saline controls levels by 14 days. Eotaxin, G-CSF,GM-CSF,IFN-γ, IL-1α, IL-1β, IL-2, IL-3, IL-4, IL-5, IL-6, IL-9, IL-10, IL-12 (p40), IL-12 (p70), MIP-1α, RANTES, TNF-α were not significantly changed at 7 and 14 days post infection. PGE_2_, an inflammatory cytokine found to be increased in pleural injury [18], was likewise increased in these pleural lavage samples versus saline control mice at 7 days post-induction of empyema (Fig. 6b).

### *pneumoniae*-mediated pleural injury is characterized by prominent visceral pleural MesoMT

**Fig. 7:**
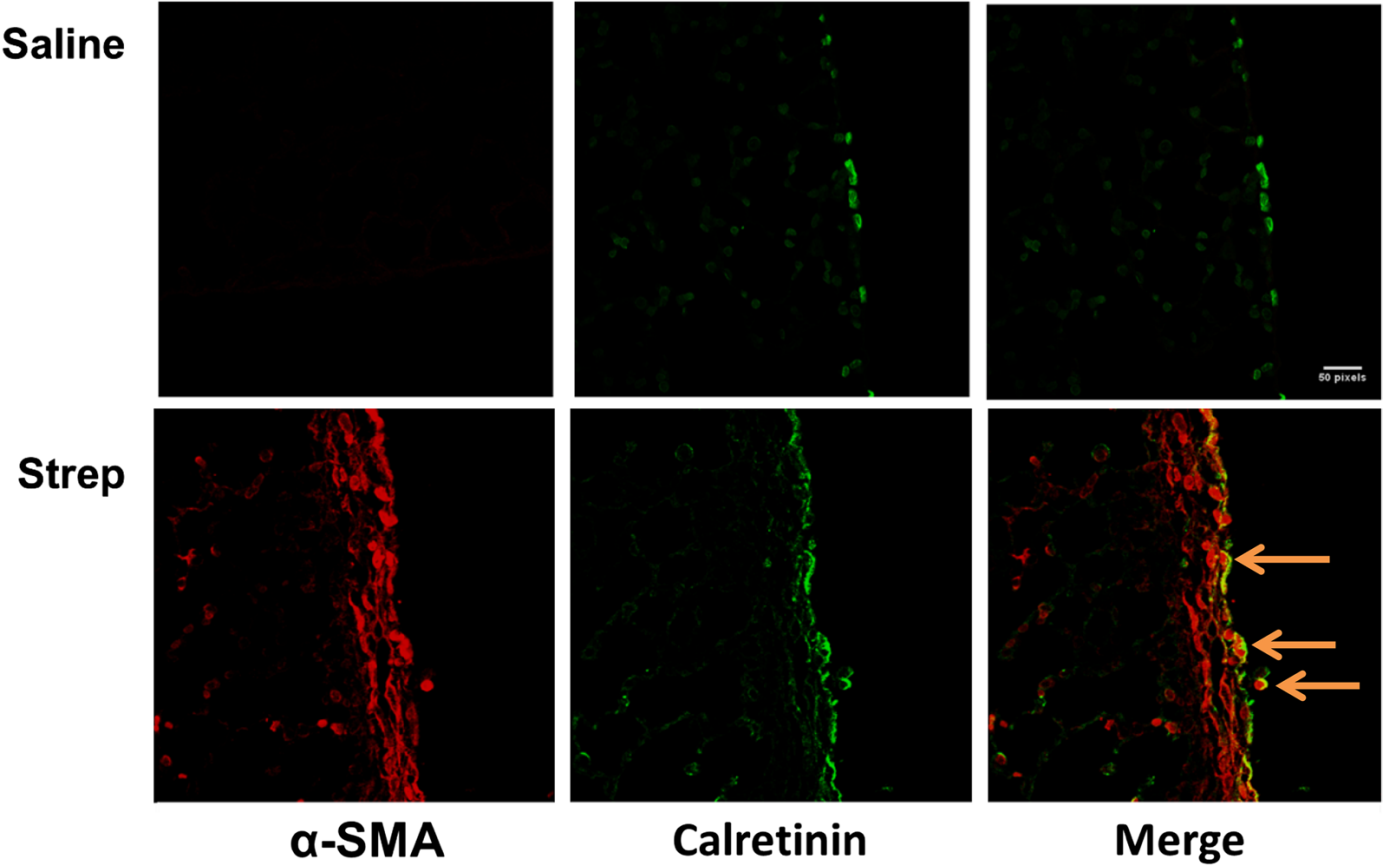
Mice with *S. pneumoniae* empyema demonstrate increased α-SMA in the mesothelium of the visceral pleura. Peripheral lung tissue sections from saline and *S. pneumoniae*-infected mice were prepared from the lungs of mice with empyema that progressed over 7 days. The sections were then probed for calretinin (*green*) and α-SMA (*red*) expression in the pleural mesothelium and observed by confocal microscopy. *Solid arrows* indicate areas of α-SMA and calretinin colocalization at the surface and within the thickened visceral pleura of *S. pneumoniae* infected mice. All images were taken at 40× magnification. Images are representative of 30 fields/slide and n = 5–6 mice/group

We previously showed that MesoMT of visceral pleural mesothelial cells likely contributes to the formation of the pleural rind associated with non-specific pleuritis in human tissues [9]. The contribution of MesoMT to pleural rind formation was also confirmed in carbon black/bleomycin-mediated pleural injury [9]. We next sought to determine the contribution of MesoMT to pleural remodeling in *S. pneumoniae*-mediated pleural injury using confocal microscopy. Saline treated mice were positive for the mesothelial protein; calretinin (green) but did not express the myofibroblast marker; α-SMA (red, Fig. 7) in the mesothelium and submesothelial tissues. Conversely, α-SMA expression was enhanced at the pleural surface and colocalized with calretinin in *S. pneumoniae*-infected mice. These findings strongly suggest that MesoMT of resident pleural mesothelial cells localizes to and contributes to the extensive pleural rind formation and remodeling associated with *S. pneumoniae*-induced pleural organization.

### PAI-1 deficiency worsens *S. pneumoniae* pleural injury at 3 days post-infection

**Fig. 8:**
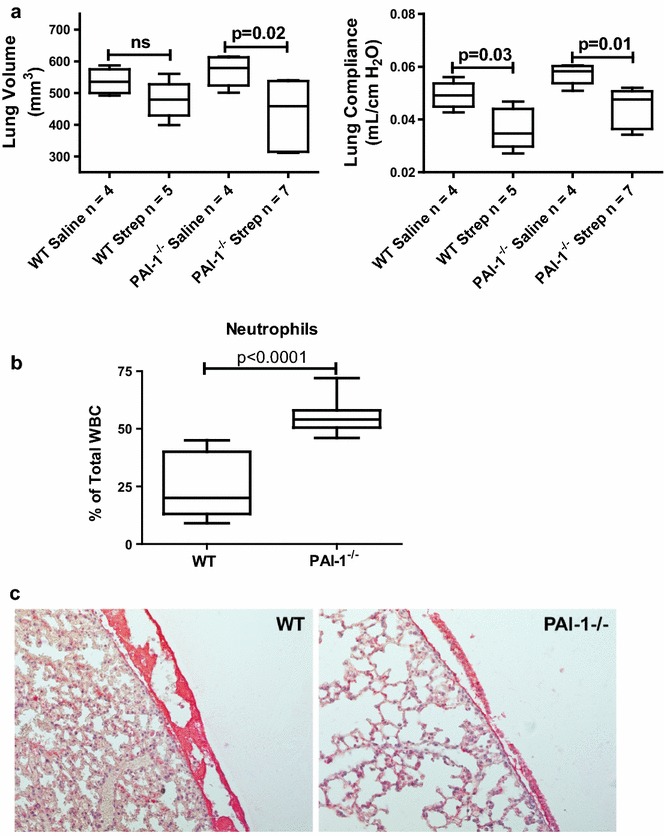
Pleural inflammation and lavage neutrophilia is increased in PAI-1 deficiency after induction of *S. pneumoniae* mediated pleural injury. Pleural injury was induced in WT and PAI-1^−/−^ mice by intrapleural injection of *S. pneumoniae* and maintained for 3 days. CT scan and pulmonary function analyses were performed 3 days after infection. **a** *S. pneumoniae* infection induced significant pleural injury in PAI-1^−/−^ mice (volume and compliance, p = 0.02 and p = 0.01, respectively). **b** Pleural lavage fluid from *S. pneumoniae* infected PAI-1^−/−^ mice contained a significantly higher percentage of neutrophils than identically treated WT mice. Neutrophil percentage was determined by differential staining and cellular differential performed at 100× oil magnification, n = 4–7/group. **c** Tissue sections from saline and *S. pneumoniae*-infected mice were prepared from the lungs of WT and PAI-1^−/−^ mice after 3 days. The sections were then stained for fibrin (ogen) (*red*) by IHC. All images were taken at 20× magnification. Images are representative of 30 fields/slide and n = 4–7 mice/group. Pleural lavages (PL) were performed on *S. pneumoniae* infected mice at 3 days

We recently showed that PAI-1 deficiency exacerbated carbon black/bleomycin-mediated pleural injury [9]. To interrogate the role of PAI-1 in the progression of *S. pneumoniae* empyema, PAI-1^−/−^ mice were used. Unlike WT mice, infected PAI-1^−/−^ mice that received antibiotic treatment demonstrated significant mortality by 4 days (data not shown) so that we could only reliably maintain these animals over 3 days. Although these animals only received 2 days of the antibiotic regimen, no bacteria were detected in the pleural washes of either WT or PAI-1 infected mice at 3 days (data not shown). While infected PAI-1^−/−^ mice demonstrated significant changes in lung volume (p = 0.02) by 3 days (Fig. 8a), trends in infected WT mice did not reach significance by 3 days. *S. pneumoniae*-infected WT and PAI-1^−/−^ mice exhibited significant changes in compliance when compared to the saline controls (p = 0.03 versus 0.01 respectively). Total WBC counts in pleural lavage were also significantly increased in *S. pneumoniae* infected WT (1.16 ± 0.24 × 10^6^ versus 11.51 ± 4.7 × 10^6^, p < 0.05) and PAI-1^−/−^ (1.46 ± 0.80 × 10^6^versus 12.7 ± 3.01 × 10^6^, n = 4–6 mice/group, p < 0.05) versus saline controls. The percentage of lavage neutrophils was also significantly increased in PAI-1^−/−^ deficient mice (p < 0.0001, Fig. 8b).

**Fig. 9:**
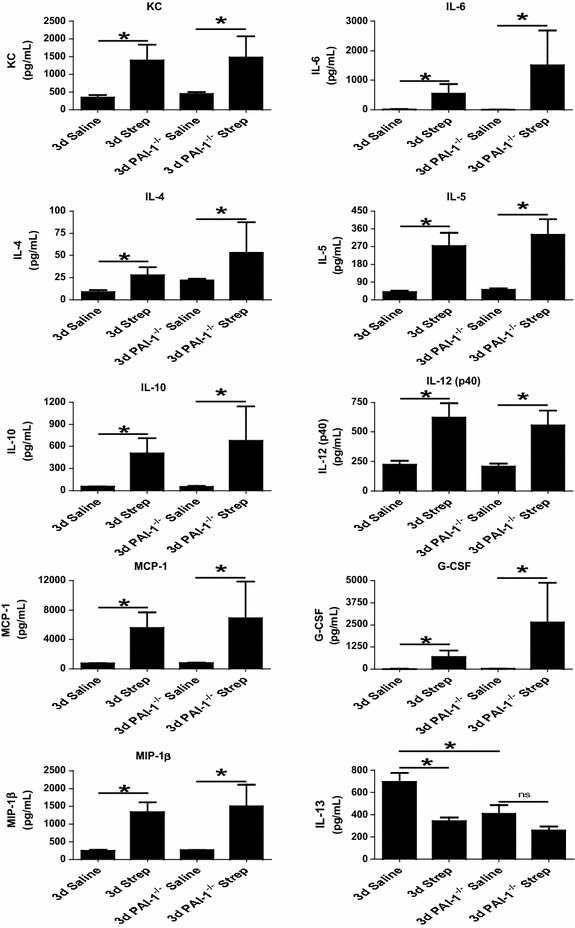
Pleural inflammation and lavage neutrophilia is increased in PAI-1 deficiency after induction of *S. pneumoniae* mediated pleural injury. Pleural injury was induced in WT and PAI-1^−/−^ mice by intrapleural injection of *S. pneumoniae* for 3 days. PLs from saline and *S. pneumoniae*-infected WT and PAI-1^−/−^ mice demonstrated significant increases in KC, IL-4, IL-5, IL-6, IL-10, IL-12, MCP-1, G-CSF, and MIP-1β * denotes P < 0.05, n = 4–7 mice/group). *S. pneumoniae* infection significantly decreased IL-13 in WT mice (p < 0.05) when compared to controls. Saline-treated PAI-1^−/−^ mice demonstrated significantly less IL-13 than similarly treated WT mice (p < 0.05). n = 4–7 mice/group

**Fig. 10:**
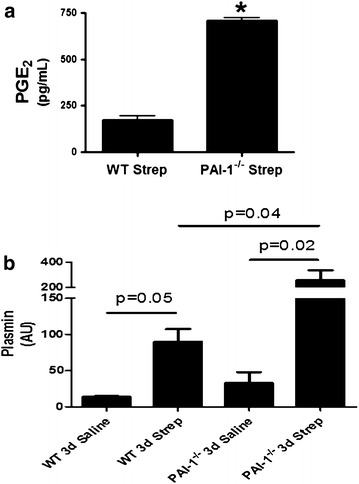
PGE_2_ and plasmin levels are increased in PAI-1 deficiency after induction of *S. pneumoniae* mediated pleural injury. Pleural injury was induced in WT and PAI-1^−/−^ mice by intrapleural injection of *S. pneumoniae* and maintained for 3 days. PGE_2_ was extracted from the PLs of saline and *S. pneumoniae*-infected mice. PGE_2_ levels were then determined by competitive ELISA. **a** PGE_2_ was significantly higher in *S. pneumoniae* infected PAI-1^−/−^ mice compared to infected WT mice at 3 days, n = 4–7 mice/group. * denotes (P < 0.05). **b** PLs from saline and *S. pneumoniae* infected WT and PAI-1^−/−^ mice were assayed for plasmin activity. *S. pneumoniae* infection significantly increased plasmin activity in WT and PAI-1^−/−^ mice (p = 0.05 and 0.02, respectively) by 3 days. *S. pneumoniae*-infected PAI-1^−/−^ mice demonstrated significantly higher plasmin activity than identically treated WT mice (p = 0.04)

Tissue sections from *S. pneumoniae*-infected WT and PAI-1^−/−^ mice were next stained for fibrin (ogen) by IHC (Fig. 8c). *S. pneumoniae* infected PAI-1^−/−^ mice demonstrated decreased pleural fibrin deposition that was readily apparent compared to WT mice treated in the same manner. Extravascular fibrin (ogen) was not detected in saline treated mice (data not shown). Tissues from 3 days infected WT and PAI-1^−/−^ mice were also stained for α-SMA to determine the extent of MesoMT. However there were no differences between infected WT and PAI-1^−/−^ mice at 3 days (data not shown).

We next determined the inflammatory mediator profile in pleural lavage of 3 days WT and PAI-1^−/−^ infected mice. WT and PAI-1^−/−^*S. pneumoniae* infected mice demonstrated significant increases in KC, IL-4, IL-5, IL-6, IL-10, IL-12, MCP-1, G-CSF and MIP-1β (Fig. 9, P < 0.05) compared to the saline controls. However there were no significant differences between WT and PAI-1^−/−^ mice, saline or infected. Conversely, IL-13 expression was significantly decreased in WT mice with infected with *S. pneumoniae* (p < 0.05) at 3 days. While IL-13 levels in saline treated PAI-1^−/−^ mice were not significantly different from *S. pneumoniae* infected PAI-1^−/−^ mice, they were significantly lower than saline treated WT mice (p < 0.05).

### PGE_2_ and plasmin are increased in PAI-1^−/−^ mice with empyema

Because PGE_2_ was significantly increased in the pleural lavages of 7 days *S. pneumoniae*-infected WT mice, we next assayed PGE_2_ levels in the pleural lavages of 3 day infected WT and PAI-1^−/−^ mice. PGE_2_ levels were significantly increased in the pleural lavages of *S. pneumoniae*-infected PAI-1 deficient mice compared to identically treated WT mice at 3 days (p < 0.05, Fig. 10a). Because plasmin has been reported to increase COX-2 and, consequently, PGE_2_ expression [19], we next assayed plasmin activity in the pleural lavages of WT and PAI-1^−/−^ mice (Fig. 10b). As anticipated, plasmin activity was significantly increased in the pleural lavage of *S. pneumoniae*-infected WT and PAI-1^−/−^ mice compared to saline controls (P = 0.05 and 0.02 respectively). Further, *S. pneumoniae* infected-PAI-1^−/−^ mice demonstrated significantly higher plasmin activity than infected WT mice (p = 0.04). These findings were confirmed by measuring the fibrinolytic potential of these fluids using FITC-labeled fibrin as previously described (data not shown) [14].

### PGE_2_ induces MesoMT

**Fig. 11:**
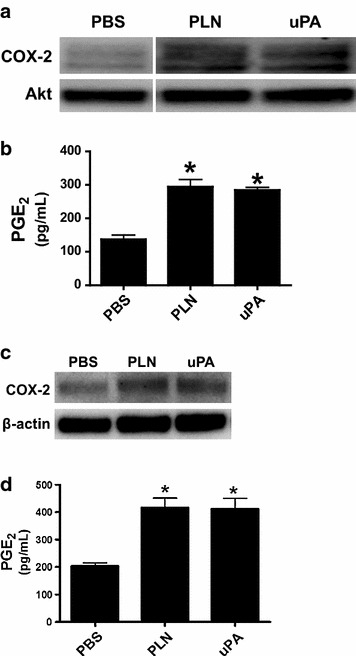
COX-2 and PGE_2_ are induced in human and murine PMCs by plasmin and uPA. **a** Serum-starved HPMCs were treated with plasmin (PLN, 7 nM) or uPA (20 nM) for 48 h. Lysates were then Western blotted for COX-2. Akt was the loading control. **b** Conditioned media of cells treated with PBS, plasmin and uPA were analyzed for PGE_2_. Plasmin and uPA significantly increased PGE_2_ expression in HPMCs (* denotes a p < 0.05 when compared to PBS treated controls. n = 3/treatment). **c** Serum-starved MPMCs were treated with murine plasmin (7 nM, PLN) and murine uPA (20 nM) for 48 h. Lysates were then subjected to western blotting for COX-2. β-actin was the loading control. **d** Conditioned media of cells treated with PBS, plasmin and uPA were probed for PGE_2_ by competitive ELISA. Plasmin and uPA significantly increased PGE_2_ expression in MPMCs (*denotes a p < 0.05 when compared to PBS treated controls)

**Fig. 12:**
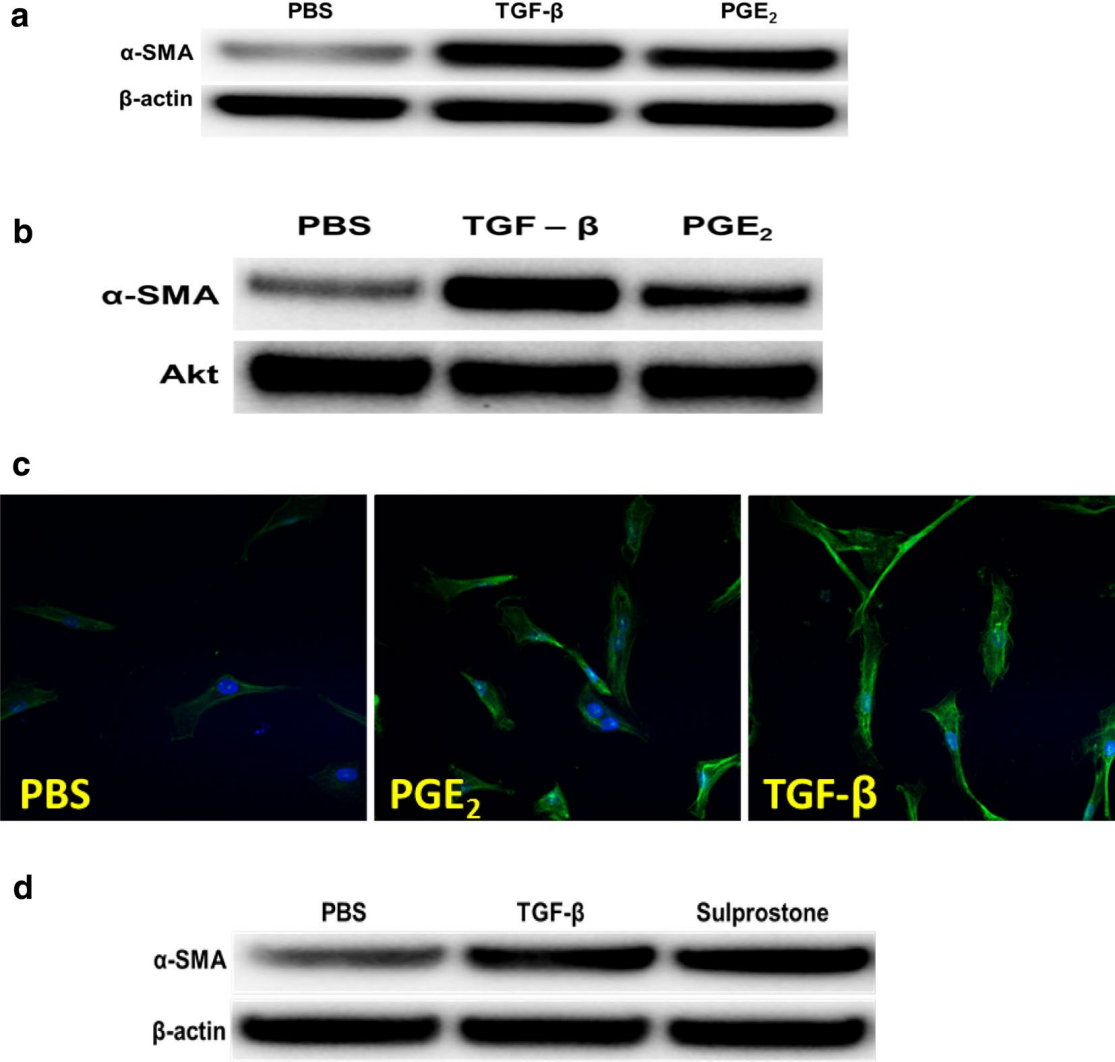
PGE_2_ induces MT of HPMCs and MPMCs. Serum-starved HPMCs were treated with PBS, PGE_2_ (1 μM ) and TGF-β (5 ng/ml) for 48 h. Cell lysates were cleared, measured and then resolved by SDS-PAGE. **a** α-SMA was increased in TGF-β and PGE_2_-treated HPMCs. β-actin was the loading control. Image is representative of three independent experiments. **b** MPMCs were serum starved for 24 h and then treated with PBS, TGF-β (5 ng/ml) or PGE_2_ (1 μM) for 48 h. Lysates were then resolved via SDS-PAGE and probed for α-SMA. Akt was the loading control. Image is representative of two independent experiments. HPMCs were seeded on glass coverslips. Cells were serum starved for 24 h and then treated with PBS, PGE_2_ or TGF-β for 48 h. **c** PGE_2_ increased α-SMA expression by HPMCs and induced changes in cell morphology indicative of MesoMT. Images were taken at 40× and are representative of 30 fields/treatment. **d** Serum-starved HPMCs were treated with PBS, TGF-β (5 ng/ml) and sulprostone (EP3 agonist, 1 µM) for 48 h. Cell lysates were cleared, measured and then resolved by SDS-PAGE. α-SMA was increased in TGF-β and sulprostone-treated HPMCs. β-actin was the loading control. Image is representative of three independent experiments

Our data suggest that increased plasmin activity in the pleural fluids of *S. pneumoniae* infected PAI-1 deficient mice may contribute to induction of PGE_2_. uPA is also increased in pleural lavage of injured PAI-1^−/−^mice [9]. Therefore, we next tested the ability of uPA and plasmin to induce cyclooxygenase-2 (COX-2) and PGE_2_ in human (H) and murine (M) PMCs. uPA- and plasmin induced COX-2 and significantly increased PGE_2_ expression by HPMCs (Fig. 11a, b, respectively) and MPMCs (Fig. 11c, d, respectively). Because PGE_2_ was increased in pleural injury and increased PGE_2_ levels occurred in pleural lavage of PAI-1^−/−^ mice, we next determined the ability of PGE_2_ to induce biomarkers of MesoMT using PMCs. PGE_2_ induced α-SMA protein expression in HPMCs and MPMCs (Fig. 12a, b respectively). TGF-β was used as a positive control in both HPMCs and MPMCs as it is an established stimulus of biomarkers of MesoMT; α-SMA in HPMCs [9, 17]. Phenotypic changes indicative of MesoMT and increased α-SMA expression were detected in PGE_2_- and TGF-β-treated HPMCs (Fig. 12c). Because PGE_2_ induced α-SMA expression, we next sought to determine which prostaglandin receptor (EP) was responsible for PGE_2_-induced α-SMA. HPMCS were treated with TGF-β and butaprost (EP2 agonist), sulprostone (EP3 agonist) or Cay10598 (EP4 agonist). β-actin was the loading control. While butaprost and Cay10598 had no effect on α-SMA expression (data not shown), sulprostone robustly induced α-SMA expression (Fig. 12d). We attempted to down-regulate the EP receptors (2–4) by siRNA transfection but were unable to sufficiently reduce EP receptor expression for our analyses.

## Discussion

Although the incidence of empyema continues to increase worldwide [1, 20], advances in its treatment remain relatively limited. Bacteriological analyses of the participants in the first Multicenter Intrapleural Sepsis Trial (MIST1) showed that the leading causes of pleural infection are Streptococcal pathogens [1, 21, 22]. Further, the leading microbial cause of community-acquired pneumonia is now *Streptococcus pneumoniae* [22]. The 12 month mortality rate associated with pulmonary *S. pneumoniae* infection is reported to be about 17 % [22]. There has been little improvement in the mortality rate due to pleural infection in the US for the last 5 decades despite therapeutic advances including vaccination to protect against *Streptococcal pneumonia* [2]. These considerations suggest an imperative to better understand mechanisms that contribute to pleural injury and identify new pathways and targets amenable to intervention. These efforts have been impeded by the lack of a survivable murine model that reliably allows investigation of the pathogenesis of pleural organization and remodeling.

Our objective was to therefore develop a murine model that enables the assessment of organizing pleural injury after acute infection. We chose to develop the model in C57BL/6 mice so that investigators in the field could use the model in genetically engineered animals that are commonly available in this strain. An alternative model of murine Streptococcal empyema was originally reported in CD1 mice by Wilkosz et al. [11]. However, the model was lethal at 48 h, which precludes analyses of subsequent changes that promote pleural organization and lung restriction. Another model has been reported in rabbits intrapleurally infected with *Pasteurella multocida* [10]. Although this model is quite robust and amenable to intervention with fibrinolytic agents, a major limitation is that the organism rarely causes pleural disease in humans.

Because pleural infection most commonly occurs as a complication of pneumonia, we initially attempted to model these circumstances through nasal delivery of *S. pneumoniae* into the lungs and monitored the development of empyema. Intranasal administration of *S. pneumoniae*-induced pneumonia with severe parenchymal inflammation and restrictive lung physiology, but sublethal doses did not reliably induce empyema, increase pleural thickening or promote pleurodesis. Further, early mortality (euthanasia required by day 2) was observed in mice with pneumonia severe enough to induce empyema. Based on histological assessments, restriction in these mice was mainly due to pneumonia rather than advanced pleural remodeling.

Because sublethal intranasal infection failed to induce pleural injury, we next administered *S. pneumoniae* directly into the intrapleural space. It is important to note intrapleural delivery models clinical pleural infection that may occur with direct pleural infection, as occurs after penetrating chest trauma, chest tube or thoracentesis-related infections or with post-surgical complications. This route of administration was characterized by the progressive accumulation of fibrinous material and increased WBCs in the pleural space by 7 days after injury. Pleural organization was also apparent by CT imaging demonstrating pleural abnormalities and by significant restrictive changes in pulmonary function by 7 days. Pleural adhesion formation and thickening and areas of pleurodesis were apparent at gross inspection and by histological analyses. Although significant decrements in lung function were observed at 3 days in infected WT mice, changes in lung volumes did not reach significance, likely attributable to modest changes in pleural organization that were found at that time. The significant decrements in lung volume and function demonstrated by infected PAI-1^−/−^ mice at 3 days appeared to be due to the presence of pleural effusions rather than advanced pleural remodeling. The presence of purulent pleural effusions and increased pleural lavage neutrophilia in PAI-1^−/−^ mice compared to WT mice demonstrates that local inflammation is worsened by PAI-1 deficiency. Although the bacterial burden in WT and PAI-1^−/−^ was comparable at 3 days, septicemia was not excluded and represents a potential determinant for increased mortality in PAI-1 deficient mice. While a range of mediators that could have contributed to increased pleural inflammation in the infected PAI-1^−/−^ mice were identified, increased pleural lavage PGE_2_ of these versus WT animals in particular likely contributed to that differential response. On the other hand, increased local elaboration of plasmin likely contributed to the decreased extravascular fibrin seen in the PAI-1^−/−^ animals with empyema (Fig. 8c), which could have impaired containment of the organisms and worsened outcomes. Lastly, it is likewise possible that local or systemic elaboration of other factors could have contributed to the increased mortality in these animals.

Antibiotic administration was required to maintain mice with empyema over 3–14 days after intrapleural inoculation. While ampicillin (100 mg/kg) is an alternative antibiotic for the treatment of *S. pneumoniae* infection, its use required subcutaneous injection every 12 h. To minimize manipulation and distress in the infected mice, we chose to use the quinolone enrofloxacin (15 mg/kg), which only required subcutaneous injection every 24 h. We did not attempt to induce empyema in mice pre-treated with antibiotics. Although antibiotic treatment cleared the bacterial infection by 3 days, decrements in lung function and pleural rind formation occurred even after clearance of viable organisms. This situation simulates the findings that can occur in complicated parapneumonic pleural effusions in patients that are predisposed to loculation. As expected, a range of inflammatory mediators were significantly increased by 3 days and tended to subside by 14 days after induction of empyema. Restrictive changes in lung function and pleural thickness persisted at 14 days after induction of Streptococcal empyema, enabling assessment of pleural remodeling at subacute stages post-infection. While we did not carry the model forward, the animals were recovering from pleural infection at 14 days and could likely be maintained for even longer periods of time to assess the resolution of pleural injury. Studies to evaluate factors that contribute to progressive remodeling after induction of empyema over longer intervals are ongoing. Future studies will also include *S. pneumoniae* strains more commonly found in the clinical setting to determine if they likewise induce comparable pleural remodeling and survival.

In our previous reports, we showed that MesoMT contributed to the increased myofibroblast population observed in human nonspecific pleuritis and in our carbon black/bleomycin pleural injury model [9, 17]. Further, MesoMT has been reported by others to be a key feature of pleural remodeling [23, 24]. Confocal analyses of tissue sections from *S. pneumoniae*-infected mice demonstrated increased α-SMA expression that colocalized within the pleural and subpleural regions, demonstrating that MesoMT occurred in WT mice by 7 days after pleural infection. Myofibroblasts from other sources such as the lung interior or fibrocytes may also be present, but the data show that mesothelial cells contribute to the process. These findings strongly suggest that pleural mesothelial cells undergo MesoMT and contribute to pleural remodeling and rind formation that occur over 2 weeks following induction of empyema.

The model is tractable for the identification of novel pathways that may condition pleural remodeling. An example of that is provided by our data showing that PGE_2_ is not only locally expressed during the organizational phase of resolving empyema, as expected, but that it can influence remodeling by stimulating MesoMT and pleural rind expansion. Plasmin was detectable during this phase and was capable of inducing PGE_2_ elaboration by murine PMCs. Plasmin and PGE_2_ potently induced biomarkers of MesoMT as did TGF-β in our analyses. We found that PGE_2_ can contribute to organization and remodeling of the visceral pleura after *S. pneumoniae*–induced injury in part by promoting MesoMT in an EP3-specific manner. The ability of plasmin itself to induce pleural rind formation is likely limited by rapid inhibition of fibrinolytic activity leading to formation of an intrapleural fibrinous transition matrix at 7 or 14 days post infection, as demonstrated by the paucity of detectable fibrinolytic activity in in lung lavage.

In summary intrapleurally administered *S. pneumoniae* induced robust pleural remodeling. The model is also characterized by progressive matrix deposition, restrictive lung disease and durable pleural remodeling. Further, the use of antibiotics allows the study of injury progression for at least 14 days and perhaps over longer periods of time. The model enables the use of genetically modified animals, as demonstrated by analyses of the effects of PAI-1 deficiency in the model. We identified enhanced susceptibility to *S. pneumoniae* infection in PAI-1^−/−^ mice and demonstrate that pleural inflammation was increased and likely due at least in part to overexpression of PGE_2_. Increased local elaboration of plasmin in the PAI-1^−/−^ mice appears to have decreased extravascular fibrin deposition, which may have impaired containment of the infection within the pleural space. Apart from these effects, other local or systemic effects including systemic sepsis could have contributed to increased mortality in the PAI-1^−/−^ mice. In the aggregate, these findings demonstrate that the model is reliably characterized by pleural organization after the induction of pleural infection and enables dissection of local alterations involved in the organizational phase of pleural injury that follows Streptococcal empyema.
